# Horizontal ecological compensation and urban resilience: mechanisms of low-carbon transformation

**DOI:** 10.3389/fpubh.2025.1583074

**Published:** 2025-09-12

**Authors:** Zhongyin An, Hongce Xiao, Weiyi Li, Hengli Wang

**Affiliations:** ^1^Office of Information Technology Development Management, South-Central Minzu University, Wuhan, China; ^2^School of Mathematics and Statistics, Zhongnan University of Economics and Law, Wuhan, China; ^3^Wuhan University of Cyber Security Preparatory Office, Wuhan, China

**Keywords:** horizontal ecological compensation, inclusive and resilient cities, environmental degradation, low-carbon transformation, industrial structure advancement, green innovation

## Abstract

**Introduction:**

This study investigates the socioeconomic impacts of horizontal ecological compensation (HEC) policies in China, focusing on their role in mitigating environmental degradation and enhancing urban resilience.

**Methods:**

We utilize panel data from 180 cities in the Yangtze and Yellow River basins (2007–2022) and construct an Inclusive and Resilient City (IRC) index. Policy effects are evaluated through a multi-period quasi-natural experiment.

**Results:**

The results indicate that HEC policies are associated with a 0.3% average increase in the IRC index, primarily driven by improvements in green innovation and industrial upgrading. These mechanisms contribute to reducing pollution-related health risks and enhance urban resilience against environmental stressors. While the average increase appears modest, it represents a meaningful improvement in urban well-being within the constraints of regional development and ecological sustainability. The robustness of these findings is confirmed through multiple tests, including parallel trend analysis and placebo tests.

**Discussion:**

This research highlights HEC policies as an innovative policy tool that balances environmental protection with socioeconomic development. To strengthen their impact, policy optimization is recommended, aiming to further alleviate the socioeconomic burdens of environmental degradation and improve public health in urban areas.

## 1 Introduction

In the face of rapid urbanization, cities are becoming vital battlegrounds for balancing economic growth, environmental sustainability, and social equity. As hubs of economic activity, urban areas are under increasing pressure to address the intertwined challenges of environmental degradation, resource depletion, and socio-economic inequality. In response to these challenges, numerous environmental and economic policies have been implemented globally to foster inclusive and resilient urban environments, aligned with the United Nations’ Sustainable Development Goals (1).

Nations worldwide have acknowledged the imperative of developing resilient cities and have actively implemented various resilience policy frameworks. For instance, the United States’ Sustainable Communities Initiative promotes integrated planning, housing-environment linkages, and regional equity (2, 3), while the European Union’s Green Deal emphasizes decarbonization, green innovation, and ecological restoration to strengthen urban resilience (4). In Japan, the Tokyo Metropolitan Territorial Plan focuses on comprehensive disaster risk reduction (5). These international approaches commonly stress cross-sector coordination, green transition, and interregional cooperation.

In contrast, China’s approach under its ecological civilization framework emphasizes Horizontal Ecological Compensation (HEC), a distinctive policy mechanism that directly links financial transfers to ecological outcomes across regions, particularly in major watersheds like the Yangtze and Yellow River basins (6). Unlike the grant-based or regulatory frameworks of the West, HEC represents a market-incentivized, performance-linked, and regional-cooperation-oriented model. This study aims to assess whether such an approach contributes to building inclusive and resilient cities, thereby offering empirical insights with potential relevance beyond China.

In China, HEC policies have emerged as a critical tool for promoting sustainable urban development. These policies incentivize regions rich in natural resources to preserve ecological balance, while encouraging economically developed areas to contribute to environmental conservation efforts. HEC is especially pertinent in ecologically sensitive regions like the Yangtze and Yellow River basins, where rapid urbanization has led to both economic growth and significant environmental degradation.

Although HEC policies are central to China’s urban and ecological development strategies, their role in promoting inclusivity and resilience remains underexplored. Most research on HEC has focused on macroeconomic or environmental outcomes, often neglecting their potential to address social equity and urban resilience. This gap is crucial, as understanding the socio-economic impacts of HEC policies is essential to ensuring that these policies contribute to truly sustainable urban development.

This study addresses this gap by investigating the relationship between HEC policies and the Inclusive and Resilient City (IRC) development in China’s Yangtze and Yellow River basins. An IRC index is constructed for 180 cities from 2007 to 2022 using the entropy-weighted Technique for Order Preference by Similarity to Ideal Solution (TOPSIS), which enables comprehensive and objective evaluation of urban resilience across multiple dimensions. To identify the causal effects of HEC policies, a multi-period Difference-in-Differences (DID) model is employed, which compares policy-treated and untreated cities over time. Furthermore, low-carbon transformation, as a pivotal pathway to achieving urban resilience, contributes to the reduction of urban environmental risks and the enhancement of urban sustainable development capabilities through industrial restructuring and green technological innovation. This study focuses on the role of HEC policies in facilitating resilient urban development, and explores the mechanisms through which low-carbon transformation contributes to this process.

Accordingly, this research aims to address the following questions: whether HEC policies significantly promote inclusive urban resilience in China’s major river basins; how low-carbon transformation, through green innovation and industrial upgrading, mediates the relationship between HEC and IRC; and whether the effects of HEC vary across regions with different levels of ecological vulnerability and economic development.

The findings aim to inform the growing discourse on ecological compensation and sustainable urbanism, offering empirical insights relevant to river-basin governance and interregional cooperation. The remainder of this paper is structured as follows: Section 2 reviews the theoretical background and formulates hypotheses on HEC and IRC. Section 3 details the empirical methodology and data. Section 4 presents the results, including robustness checks and heterogeneity analysis. Finally, Section 5 concludes the study.

## 2 Literature review and hypothesis development

This section provides a review of the relevant literature and builds the theoretical foundation for the study. It is organized into two parts: section 2.1 reviews existing research on horizontal ecological compensation (HEC) and inclusive urban resilience (IRC); section 2.2 develops specific research hypotheses based on identified gaps and conceptual linkages.

### 2.1 Literature review

Ecological compensation policies are integral to environmental governance, playing a pivotal role in addressing both environmental degradation and socio-economic challenges (7, 8). In particular, HEC policies are institutional innovations that promote sustainable development by fostering cooperation among regions with interconnected ecological interests (9, 10). These policies are key to advancing ecological civilization, particularly in areas facing rapid urbanization and environmental stress (11, 12). By incentivizing ecological protection and ensuring equitable distribution of environmental resources, HEC policies contribute to the development of sustainable governance models that address both economic and environmental challenges (13–16).

The concept of Inclusive and Resilient Cities is a comprehensive framework that integrates economic development, social equity, and environmental sustainability (17, 18). IRC emphasizes economic growth that provides equal opportunities for all, ensuring that the benefits of development are widely shared (19, 20). In addition to inclusivity, IRC focuses on urban resilience, enabling cities to navigate and thrive amidst various challenges such as climate change and socio-economic disruptions (21). The integration of economic, social, and environmental systems in IRC highlights the importance of fostering urban environments that are both equitable and adaptable (22, 23).

Despite the growing literature on resilience and inclusivity in cities, the connection between HEC policies and IRC remains underexplored. Most studies on IRC focus on measuring resilience and inclusivity through frameworks such as the Disaster Resilience Framework (24) and the Urban Basic Strength Index (25). Factors such as climate change, green infrastructure, emergency response capability, artificial intelligence and economic development are commonly analyzed in relation to IRC (26–31). The intersection between HEC and IRC, however, remains underexplored.

HEC policies represent a strategic mechanism for fostering IRC by balancing economic growth with environmental protection. Research on HEC highlights its environmental benefits, including improved water governance, pollution control, and ecosystem restoration (32, 33). Additionally, HEC supports labor division and industrial structure adjustment, contributing to sustainable urban development (34). These policies can help cities achieve balanced economic growth and ecological sustainability, creating safer, greener, and more inclusive urban environments (35, 36).

However, HEC policies are not without challenges. Some studies suggest that they may hinder technological progress in upstream regions by negatively impacting enterprise profitability, scale, human capital, and management efficiency (36). Furthermore, concerns have been raised that HEC may exacerbate disparities between developed and less-developed regions, creating uneven economic benefits from environmental governance (37). Understanding these dynamics is crucial for evaluating whether HEC policies can support inclusive and resilient urban development without reinforcing existing inequalities.

Despite substantial attention given to the environmental and macroeconomic benefits of ecological compensation policies (20, 30, 34, 38–40), limited research has examined how HEC policies affect inclusivity and resilience in urban environments. Existing studies have largely focus on ecological and economic dimensions, often neglecting the transformative potential of HEC for urban socio-economic structures and equitable development. This reveals a critical knowledge gap regarding the extent to which HEC policies contribute to fostering Inclusive and Resilient Cities (IRC), particularly within the context of China’s rapid urbanization.

To fill this gap, this study makes several contributions. First, it constructs a multidimensional IRC index using the entropy-weighted TOPSIS method, enabling a comprehensive and quantitative assessment of urban resilience. Second, it employs a multi-period DID method to identify the causal effects of HEC policy implementation across time and space. Third, it explores heterogeneity in policy impacts, revealing stronger effects in upstream and underdeveloped areas. Finally, the study situates the findings within the broader context of international ecological governance and urban resilience strategies, thereby enhancing the relevance of China’s HEC experience to global urban sustainability debates. By examining the influence of HEC policies on IRC in the Yangtze and Yellow River basins, this research advances understanding of how ecological compensation mechanisms can promote not only environmental sustainability, but also inclusive and resilient urban development.

### 2.2 Research hypothesis

Based on the theoretical background and empirical findings discussed earlier, this subsection formulates the study’s main hypotheses. The proposed hypotheses aim to clarify the potential causal pathways between HEC policies and IRC outcomes, while considering moderating mechanisms such as green innovation and industrial upgrading.

#### 2.2.1 Inclusive and resilient cities and HEC

Inclusive and resilient cities are crucial for advancing urban areas toward greener, more inclusive, and sustainable development (2, 18). HEC policies represent a significant institutional innovation supporting the practical realization of these principles. Although research on IRC has explored its drivers and impacts, findings remain inconclusive. Previous studies have examined various factors, such as energy efficiency, sectoral development, and environmental regulation, to better understand IRC outcomes (30).

HEC policies, an economic strategy designed to enhance ecological protection and social equity, directly benefits cities by reducing carbon emissions and increasing residents’ incomes (32, 41, 42). As a form of innovative environmental regulation, HEC policies help optimize labor distribution within urban agglomerations and serve as a critical tool for sustainable urban development (43).

Most scholars agree that HEC policies can alleviate poverty among rural households and promote inclusive regional economic development (33, 39). Research has further demonstrated that ecological compensation stimulates regional economic growth and reduces disparities by influencing capital growth rates (44, 45). These policies also promote poverty reduction and the narrowing of social development gaps (46–49). As a crucial environmental policy, HEC policies directly enhance the urban ecological environment and bolsters urban risk resilience (50–52, 93).

Given these findings, this study proposes the following hypothesis:

*H1*: HEC policies can generally increase the level of IRC.

#### 2.2.2 Pathways of HEC policies on inclusive and resilient cities through low-carbon transformation

Following the implementation of HEC policies for the Yangtze and Yellow Rivers, significant positive effects have been observed in water environment management (33). The linkage between water quality assessments and local government performance evaluations (54, 55) creates strong incentives for governments to meet environmental standards, which in turn drive the low-carbon transformation of industrial structures. Local governments must meet rigorous water quality standards to receive ecological compensation from downstream areas (11, 33). Stronger environmental regulations in these areas have also contributed to low-carbon transformation of industrial structures, leading to shifts away from high-carbon-emission industries and toward more sustainable sectors (56). This process unfolds through several pathways crucial for achieving urban resilience through low-carbon development.

Relocation and Restructuring for Low-Carbon Transition: Stricter environmental regulations increase operational costs for high-carbon-emission firms, often leading to their relocation to regions with more lenient standards (15, 57–62). This contributes to regional industrial low carbonization.

Industrial Shifts toward Low-Carbon Sectors: The heightened regulatory intensity creates market barriers for high-carbon-emission firms while providing opportunities for cleaner industries, such as the service sector and renewable energy industries, to thrive (56, 63).

Low-Carbon Technological Innovation: High-carbon-emission firms may respond to regulatory pressures by investing in green technologies and research (33, 64), advancing the overall industrial structure toward a low carbon economy (65). As this contributes to the overall improvement of urban industrial frameworks toward low carbonization (34, 66). Based on these pathways, the study hypothesizes:

*H2*: The advancement of low-carbon industrial structure positively moderates the impact of HEC policies on IRC.

HEC policies also promote low-carbon technology innovation by encouraging firms to invest in climate mitigation technologies and green productivity (53, 67). This investment enhances the total factor productivity of green energy, contributing to urban resilience and broader low-carbon development objectives (35, 44, 68, 69). Recent empirical evidence demonstrates that both mandatory regulations and voluntary management certifications can improve innovation performance in firms, further strengthening green technological innovation development (70, 71). Therefore, this study proposes Hypothesis 3:

*H3*: HEC policies enhance IRC by promoting green technological innovation.

#### 2.2.3 Diverse regional impacts: HEC’S influence on IRC

HEC policies provide financial benefits to upstream areas of river basins through transfer payments (54). These compensation funds directly boost the residents’ incomes, facilitate the rural labor transfer, create employment opportunities, and stimulate economic development (37, 54, 72). Consequently, the impact of HEC policies on IRC is more pronounced in upstream areas and regions with lower levels of marketization (63, 73), where economic structures are more dependent on ecological compensation funds.

In contrast, downstream regions with higher levels of marketization and stronger economic foundations may experience relatively smaller impacts from HEC policies on IRC (63). Therefore, this study proposes Hypothesis 4:

*H4*: HEC policies exhibit distinct regional effects in promoting IRC, with stronger impacts observed in upstream and less marketized regions.

**Figure 1:**
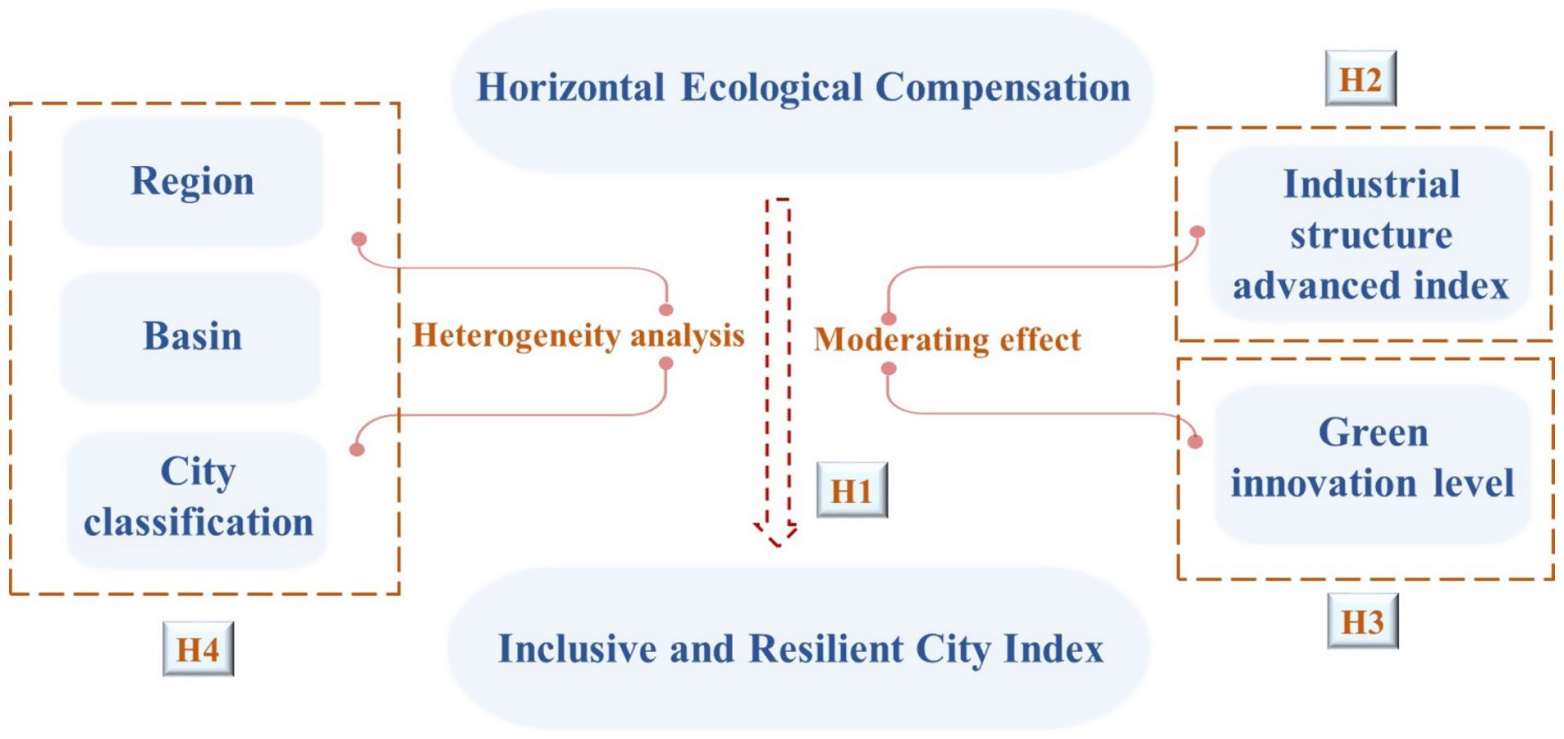
HEC policy mechanisms and effects on IRC.

Figure 1 illustrates the relationships between the four hypotheses and outlines how HEC policies influence the mechanisms of IRC.

## 3 Methodology and variables

This section introduces the empirical strategy and data used to assess the impact of HEC policies on IRC. It outlines the econometric framework, sample selection, and construction of the IRC index, followed by descriptions of the core and control variables.

### 3.1 Multi-temporal DID baseline regression model

To rigorously assess the impact of HEC policies on IRC, a multi-period DID regression model is employed (Equation 1). This method enables a detailed evaluation of the policy’s effects, accounting for the phased rollout of HEC policies initiatives across pilot cities in the Yangtze and Yellow River basins.


(1) $$\begin{array}{c} \mathrm{IR}C_{\mathrm{it}}=\alpha +\text{βHEC}\_\text{Polic}y_{\mathrm{it}}+\text{γControl}\_\mathrm{Va}r_{\mathrm{it}}\\ +\text{CiFE}+\text{YearFE}+\varepsilon_{\mathrm{it}} \end{array}$$

Among them, i and *t* represent the region and year, respectively. The variable *IRC* denotes the value of the IRC index. The term HEC_Policy captures the effect of the horizontal eco-compensation policy, while $\text{Control}\_\mathrm{Va}r_{\mathrm{it}}$ includes a set of control variables. CiFE and YearFE represent fixed effects for the city and year, respectively, and ε is the random error term. The estimation coefficient β measures the average change in the IRC index before and after the implementation of the HEC policy pilot.

### 3.2 Moderating effect model

Building on the above analysis, this study aims to assess how HEC policies IRC through a moderating effect test (Equations 2, 3):


(2) $$\begin{array}{c} \mathrm{IR}C_{\mathrm{it}}=\alpha_{2}+\beta_{2}\mathrm{HEC}\_\text{Polic}y_{\mathrm{it}}+\eta_{2}\mathrm{HEC}\_\text{Polic}y_{\mathrm{it}}\times \mathrm{AI}S_{\mathrm{it}}\\ +\theta_{2}\mathrm{AI}S_{\mathrm{it}}+\gamma_{2}\text{Control}\_\mathrm{Va}r_{\mathrm{it}}+\text{CiFE}+\text{YearFE}+\varepsilon_{\mathrm{it}} \end{array}$$

(3) $$\begin{array}{c} \mathrm{IR}C_{\mathrm{it}}=\alpha_{3}+\beta_{3}\mathrm{HEC}\_\text{Polic}y_{\mathrm{it}}+\eta_{3}\mathrm{HEC}\_\text{Polic}y_{\mathrm{it}}\times \mathrm{GI}L_{\mathrm{it}}\\ +\theta_{3}\mathrm{GI}L_{\mathrm{it}}+\gamma_{3}\text{Control}\_\mathrm{Va}r_{\mathrm{it}}+\text{CiFE}+\text{YearFE}+\varepsilon_{\mathrm{it}} \end{array}$$

Specifically, AIS denotes the degree of advancement of industrial structure, and *GIL* represents the level of green innovation. If $\eta_{2}$ show significance and both $\beta_{2}$ and $\eta_{2}$ share the same sign, it indicates that an advanced industrial structure enhances the impact of HEC policies on IRC. Similarly, If $\eta_{3}$ is significant and both $\beta_{3}$ and $\eta_{3}$ are of the same sign, the level of green innovation will strengthen the influence of HEC policies on IRC.

### 3.3 Entropy-weighted TOPSIS method

The entropy weight TOPSIS method is an effective tool for addressing complex multi-attribute decision-making problems (74). This approach combines the objective weighting strength of the entropy weight method with the comprehensive evaluation capabilities of the TOPSIS method (75). The entropy weight method, grounded in information entropy theory, calculates weights based on the variability of each evaluation index, reducing the influence of subjective bias in weight assignment (76).

Considering that the entropy weight TOPSIS method, compared with PCA, is more suitable for scenarios that require ranking and supports multi-dimensional quantitative evaluation of policies. PCA is suitable for extracting a few core dimensions from multiple indicators. Since this paper needs to determine the level of urban resilience and evaluate the impact of policies on it, choosing the entropy weight TOPSIS method is conducive to objectively ranking multiple indicators and emphasizing the interpretability of the weights. Summarizing the existing studies, it can also be found that when scholars measure urban resilience, they often use the entropy weight TOPSIS method to evaluate the levels of urban resilience in different years and regions (77, 91).

To evaluate the level of inclusive and resilient urban development, this study constructs a composite IRC index using the entropy-weighted TOPSIS method, which effectively combines objective weighting and multi-criteria ranking to address complex indicator systems.

#### 3.3.1 Step 1: indicator system and data matrix construction

An initial decision matrix was constructed based on the IRC index system, utilizing 16 years of data from 180 cities, resulting in 2,880 assessment objects and 26 indicators. The subsequent step involved standardizing the matrix, as outlined below (Equation 4).


(4) $$Z=(\begin{array}{cccc} z_{11} & z_{12} & \cdots & z_{1m}\\ z_{21} & z_{22} & \cdots & z_{2m}\\ \vdots & \vdots & \ddots & \vdots \\ z_{n1} & z_{n2} & \cdots & z_{\mathrm{nm}} \end{array})$$

#### 3.3.2 Step 2: data standardization

To eliminate dimensional inconsistency, the matrix is standardized. For positive indicators (Equation 5):


(5) $$r_{\mathrm{ij}}=\frac{Z_{\mathrm{ij}}-\min(Z_{j})}{\max(Z_{j})-\min(Z_{j})}$$

For negative indicators (Equation 6):


(6) $$r_{\mathrm{ij}}=\frac{\max(Z_{j})-Z_{\mathrm{ij}}}{\max(Z_{j})-\min(Z_{j})}$$

This produces a normalized matrix $R=r_{\mathrm{ij}}$.

#### 3.3.3 Step 3: entropy weight calculation

Entropy weights are computed to reflect the degree of variation for each indicator:

Proportion of indicator j for city i (Equation 7):


(7) $$p_{\mathrm{ij}}=\frac{r_{\mathrm{ij}}}{\sum_{i=1}^{n}r_{\mathrm{ij}}}$$

Entropy value for indicator j (Equation 8):


(8) $$e_{j}=-k\sum_{i=1}^{n}p_{\mathrm{ij}}\ln(p_{\mathrm{ij}}),k=\frac{1}{\ln(n)}$$

Difference coefficient (Equation 9):


(9) $$d_{j}=1-e_{j}$$

Final weight (Equation 10):


(10) $$w_{j}=\frac{d_{j}}{\sum_{j=1}^{n}d_{j}}$$

This approach ensures objective, data-driven weights, avoiding subjectivity in indicator importance assignment.

#### 3.3.4 Step 4: TOPSIS scoring

A weighted normalized matrix Z is obtained by Equation 11:


(11) $$Z_{\mathrm{ij}}=w_{j}\cdot r_{\mathrm{ij}}$$

The positive ideal solution (PIS) and negative ideal solution (NIS) are identified for each indicator (Equation 12):

Define PIS:


(12) $$Z^{+}=(Z_{1}^{+},Z_{2}^{+},\cdots ,Z_{m}^{+})=(\begin{array}{l} \max\{z_{11},z_{21},\cdots ,z_{n1}\},\\ \max\{z_{12},z_{22},\cdots ,z_{n2}\},\\ \max\{z_{1m},z_{2m},\cdots ,z_{\mathrm{nm}}\} \end{array})$$

Defined NIS (Equation 13):


(13) $$Z^{-}=(Z_{1}^{-},Z_{2}^{-},\cdots ,Z_{m}^{-})=(\begin{array}{l} \min\{z_{11},z_{21},\cdots ,z_{n1}\},\\ \min\{z_{12},z_{22},\cdots ,z_{n2}\},\\ \min\{z_{1m},z_{2m},\cdots ,z_{\mathrm{nm}}\} \end{array})$$

Define the distance between the i th (i=1,2,⋯,n) rating object and the maximum value:

The Euclidean distance between the i th (i=1,2,⋯,n) to the PIS (Equation 14) and NIS (Equation 15) is then calculated:


(14) $${D_{i}}^{+}=\sqrt{\sum_{j=1}^{m}w_{j}{\left(Z_{j}^{+}-z_{\mathrm{ij}}\right)}^{2}}$$

(15) $${D_{i}}^{-}=\sqrt{\sum_{j=1}^{m}w_{j}{\left(Z_{j}^{-}-z_{\mathrm{ij}}\right)}^{2}}$$

The relative closeness to the ideal solution is computed as Equation 16:


(16) $$S_{i}=\frac{D_{i}^{-}}{D_{i}^{+}+D_{i}^{-}}$$

Normalized the score $\left(\sum_{i=1}^{n}\tilde{S_{i}}=1\right)$ (Equation 17):


(17) $$\tilde{S_{i}}=\frac{S_{i}}{\sum_{i=1}^{n}S_{i}}$$

#### 3.3.5 Step 5: interpretation

The entropy-TOPSIS method enables a transparent and objective synthesis of multiple dimensions into a single IRC index. By integrating entropy-based weighting and the TOPSIS ranking procedure, this approach ensures robust comparability across time and space, while mitigating subjective bias. The resulting index is used as the key dependent variable in subsequent regression analysis.

Using normalized scores, this study evaluates 2,880 objects comprehensively. The entropy weights for each indicator were first calculated based on their information entropy, reflecting the degree of dispersion across cities and years. These weights were then applied to the standardized decision matrix to construct a weighted performance matrix. Subsequently, the TOPSIS method was used to compute the relative closeness of each city-year observation to the ideal solution, which serves as the IRC index. By integrating objective weight determination and multi-criteria evaluation, the entropy-TOPSIS approach enhances the accuracy, comparability, and reliability of the resilience assessment.

### 3.4 Variables

This subsection defines the key variables used in the empirical analysis, including the dependent variable (IRC index), core explanatory variables (HEC policy implementation), moderating variables (green innovation and industrial upgrading), and control variables. The measurement rationale for each is provided below.

#### 3.4.1 Dependent variable: IRC

Assessment index systems focusing on urban resilience have been widely applied in numerous studies. For example, the Resilient City Index developed by Economist Impact (78) evaluates 25 global cities based on four dimensions: critical infrastructure, environmental, socio-institutional, and economic factors. Ribeiro et al. (79) classified urban resilience into five dimensions: natural, economic-social, physical, and institutional. Similarly, Xun et al. (80) constructed a resilience assessment model using the TOPSIS method, incorporating 28 factors across economic, social, community facilities, and ecological environment aspects.

Other studies integrate urban characteristics with resilience components. Burton (81) and Sharifi (82) examined cities’ resilience, sensitivity, and adaptability across social, political, economic, and hydrological contexts. Ouyang (83) evaluated urban infrastructure resilience across three phases: resistance, absorption, and recovery. Zhang et al. (84) f highlighted the initial response of physical systems followed by societal feedback during natural disasters.

Overall, natural, social, economic, and infrastructure dimensions are frequently used in urban resilience assessments. However, many frameworks take a narrow focus, often overlooking the need for inclusive urban development. To address this gap, a comprehensive approach is required, one that integrates inclusiveness with ecological, economic, social, and infrastructural aspects of urban resilience.

Responding to this need, and drawing on methodologies from Cheek and Chmutina (24) and Wojewnik-Filipkowska et al. (25), this paper establishes a scientifically grounded evaluation index system for inclusive and resilient cities. The framework includes 26 factors across five core dimensions: economy, production, ecology, infrastructure, and organizational systems. Detailed indicators for each dimension are outlined in Table 1.

**Table 1:** IRC index system.

Level 1	Level 2	Level 3	Short name
Inclusive and Resilient Cities	Economy Resilience	GDP per capita (Yuan/person)	PGDP
		Gross regional product (ten thousand Yuan)	GDP
		Regional Production Index of Secondary industry (previous year = 100)	SI
		Regional Production Index of Tertiary industry (last year = 100)	TI
		Per capita Disposable income of urban residents (Yuan/person)	UDI
	Production Resilience	Energy consumption per unit of output value (tons/10,000 yuan) = total energy consumption/GDP	EC
		Carbon dioxide emissions per unit of output value (tons/10,000 yuan)	CDE
		Industrial sulfur dioxide emissions per unit of output value (tons/10,000 yuan)	ISDE
		Industrial smoke and dust emissions per unit of output value (tons/10,000 yuan)	IDE
		Industrial wastewater discharge per unit of output value (tons/10,000 yuan)	IWD
	Ecology Resilience	Green space rate (%)	GS
		Green coverage rate of built-up area (%)	BAGC
		Per capita green park area (square meters)	GPA
		Wastewater discharge compliance rate (%)	WDS
		Comprehensive utilization rate of solid waste (%)	CUSW
	Infrastructure Resilience	Water resources per capita (m3/person)	CWR
		Per capita road area (SQM/person)	RA
		Drainage pipe density (km/km ^2^)	DPD
		Gas penetration rate (%)	GPR
		Number of mobile phone households (households)	MPH
	Organizational Resilience	Education expenditure (ten thousand yuan)	EA
		Number of health technicians per 1,000 population (persons)	HT
		Number of beds in medical and health institutions per 1,000 population (sheets)	BMH
		Number of participants in urban basic endowment Insurance (10,000)	UBEN
		Number of urban basic medical insurance participants (10,000)	UBMI
		Social security expenditure as a percentage of government expenditure (%)	SSE

#### 3.4.2 Explanatory variables: HEC policies

This study employs a quasi-natural experiment to assess the impact of the HEC policy. Cities that implemented the HEC policy are classified as the experimental group (coded as 1), while those that did not are designated as the control group (coded as 0). Time dummy variables are used to distinguish periods before (coded as 0) and after (coded as 1) the policy implementation. Given that the HEC policy was introduced in stages across different cities, the time dummy variable reflects the specific timing of the policy rollout in each city.

#### 3.4.3 Control variables

IRC are influenced by various natural, economic, and political factors. To minimize errors from missing variables and enhance the reliability of our analysis, this study adopts the research approach outlined by Schintler and McNeely (29), Wang and Chen (30), and Zhou et al. (31). Four control variables are included in the model: Urban Openness, Government Macroeconomic Control capacity, Fiscal Decentralization, and Financial Development level.

#### 3.4.4 Moderating variables

Building upon the mechanistic analysis presented in Section 2, this paper examines how HEC policies influence IRC by enhancing green innovation and advancing industrial structures. According to Yang (45) and Li (85), green innovation is measured by the number of green invention patents obtained by each city. In this study, the level of green innovation is defined as the natural logarithm of the total number of green patent applications plus one.

To assess the advancement of industrial structures, a combined methodological approach proposed by Xiang (86) and Dong (87) is adopted. The ratio of the output value of the tertiary industry to that of the secondary industry serves as the measure of industrial upgrading. An increasing ratio indicates a shift toward a service-oriented economy, reflecting the ongoing enhancement of the industrial structure. However, it is noteworthy that within the context of Horizontal Ecological Compensation, this industrial structural upgrading is not merely characterized by an increased proportion of the service sector, but more importantly, by a transition toward a greened industrial structure.

#### 3.4.5 Data source and study area

This study analyzes data from 178 prefecture-level cities and 2 municipalities in the Yangtze and Yellow River basins, covering the period from 2007 to 2022. These regions were selected because they are key pilot areas for HEC policy implementation and represent critical zones for ecological compensation and green development in China (63). The cities were chosen based on policy relevance and data availability, rather than random sampling, to ensure alignment with the research objectives. Although this regional focus may limit generalizability to all Chinese cities, it enables a detailed examination of the mechanisms through which HEC policies affect IRC. The data were sourced from the China Urban Statistical Yearbook, China Energy Statistical Yearbook, China Environmental Statistical Yearbook, China Environmental Situation Bulletin, and China Water Resources Bulletin and missing values were addressed using interpolation. City-level details are provided in Appendix 1, and variables are listed in Table 2.

**Table 2:** Data source.

Variable	Short name	Data source	Data Website
Inclusive and resilient cities	IRC	China city statistical year book & China statistical year book on environment	https://cnki.ctbu.edu.cn/CSYDMirror/area/Yearbook/Single/N2021050059?z=D26 & https://www.stats.gov.cn/sj/ndsj/
Green Innovation Level	GIL	Chinese research data services platform	https://www.las.ac.cn/front/dataBase/detail?id=45d5100e2586517c068ee112cdeb7d3a
Advancing Industrial Structures	AIS	China Industrial Enterprises Database	https://www.shujuku.org
Urban Openness	UO	China city statistical yearbook	https://cnki.ctbu.edu.cn/CSYDMirror/area/Yearbook/Single/N2021050059?z=D26
Government Macroeconomic Control capacity	GMC	China city statistical yearbook	
Fiscal Decentralization	FD	China city statistical yearbook	
Financial Development level	FD	China city statistical yearbook	

## 4 Result

This section presents the empirical results derived from the baseline regression models. It examines the direct effects of HEC policies on IRC, followed by a series of robustness checks and heterogeneity analyses to ensure the credibility and consistency of the findings.

### 4.1 Measurement and analysis of IRC

This study evaluates the IRC scores of 180 cities in the Yangtze and Yellow River basins from 2007 to 2022, following the methodology outlined by the indicators of urban resilience (88). Using the entropy weight TOPSIS method, which assigns equal importance to each IRC aspect, each indicator contributes 20% to the overall score. Adjustments based on these weights are presented in Table 3.

**Table 3:** IRC weight proportions.

Level 1	Level 2	Level 3	Weight
Inclusive and resilient cities	Economy resilience	GDP per capita (Yuan/person)	0.025
		Gross regional product (ten thousand Yuan)	0.083
		Regional Production Index of Secondary industry (previous year = 100)	0.391
		Regional Production Index of Tertiary industry (last year = 100)	0.009
		Per capita Disposable income of urban residents (Yuan/person)	0.020
	Production resilience	Energy consumption per unit of output value (tons/10,000 yuan) = total energy consumption /GDP	0.005
		Carbon dioxide emissions per unit of output value (tons/10,000 yuan)	0.006
		Industrial sulfur dioxide emissions per unit of output value (tons/10,000 yuan)	0.002
		Industrial smoke and dust emissions per unit of output value (tons/10,000 yuan)	0.076
		Industrial wastewater discharge per unit of output value (tons/10,000 yuan)	0.020
	Ecological resilience	Green space rate (%)	0.083
		Green coverage rate of built-up area (%)	0.003
		Per capita green park area (square meters)	0.001
		Wastewater discharge compliance rate (%)	0.001
		Comprehensive utilization rate of solid waste (%)	0.026
	Infrastructure resilience	Water resources per capita (m3/person)	0.042
		Per capita road area (SQM/person)	0.055
		Drainage pipe density (km/km ^2^)	0.009
		Gas penetration rate (%)	0.053
		Number of mobile phone households (households)	0.052
	Social resilience	Education expenditure (ten thousand yuan)	0.011
		Number of health technicians per 1,000 population (persons)	0.001
		Number of beds in medical and health institutions per 1,000 population (sheets)	0.001
		Number of participants in urban basic endowment Insurance (10,000)	0.006
		Number of urban basic medical insurance participants (10,000)	0.005
		Social security expenditure as a percentage of government expenditure (%)	0.014

**Figure 2:**
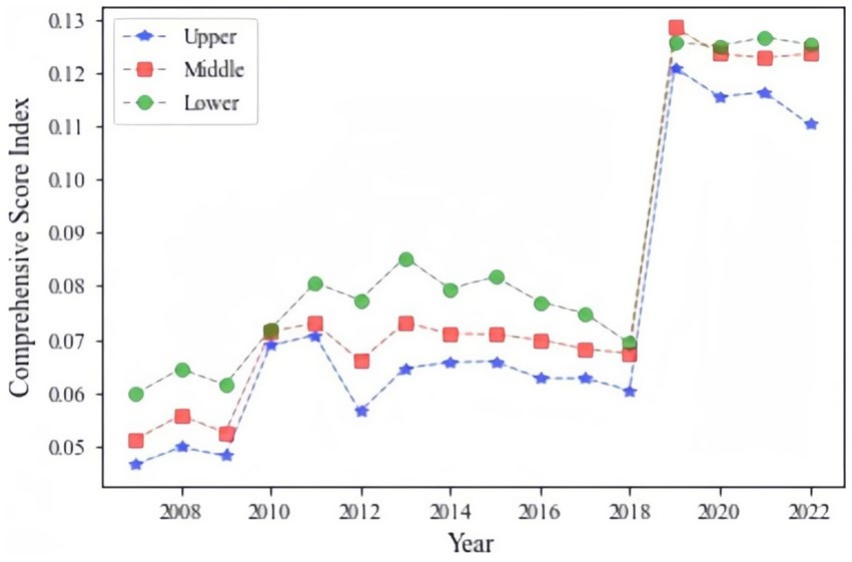
Trend of average comprehensive evaluation score.

The analysis reveals that the average urban resilience score is 0.081, indicating a generally low level of resilience across Chinese cities during the study period. However, as shown in Figure 2, the scores demonstrate an upward trend over time, with cities in the upper and middle reaches of the Yellow and Yangtze River basins showing higher resilience levels compared to those in the lower reaches.

### 4.2 Regression results analysis

This study employs a multi-stage DID model to analyze the impact of HEC policies on the levels of IRC in the Yangtze and Yellow River basins. The basic regression results are presented in Table 4. In column (1), after accounting for city and year effects, the DID coefficient is 0.003, statistically significant at the 1% level. This significance persists in column (2) even after adding control variables, indicating that the HEC policy effectively promotes IRC, supporting Hypothesis H1.

**Table 4:** Regression results.

Variable	(1)	(2)	(3)	(4)	(5)	(6)	(7)
	IRC	IRC	ENR	PR	ELR	IR	SR
did	0.003***	0.003***	0.002***	0.002	0.044***	0.007***	−0.001
	(3.155)	(2.892)	(2.601)	(0.290)	(3.647)	(3.997)	(−0.689)
UO		−0.070**	−0.043*	0.261	0.209	−0.044	−0.344***
		(−2.095)	(−1.687)	(1.149)	(0.558)	(−0.795)	(−6.093)
GMC		−0.020***	−0.001	−0.009	0.047	−0.009	−0.084***
		(−2.619)	(−0.231)	(−0.167)	(0.544)	(−0.673)	(−6.507)
FD		−0.002	0.001	0.074**	−0.039	−0.003	−0.040***
		(−0.410)	(0.244)	(2.532)	(−0.808)	(−0.454)	(−5.486)
FDL		0.006***	−0.003*	0.041***	0.003	−0.010***	0.020***
		(3.104)	(−1.941)	(2.933)	(0.116)	(−2.812)	(5.873)
_cons	0.113***	0.116***	0.018***	0.631***	0.912***	0.016**	0.046***
	(29.245)	(23.959)	(4.868)	(19.209)	(16.804)	(1.964)	(5.691)
Year fix	Yes	Yes	Yes	Yes	Yes	Yes	Yes
City fix	Yes	Yes	Yes	Yes	Yes	Yes	Yes
*N*	2,879	2,879	2,879	2,879	2,879	2,879	2,879
*R* ^2^	0.800	0.801	0.462	0.880	0.606	0.476	0.743
Adj. *R*^2^	0.786	0.787	0.422	0.871	0.577	0.437	0.724

Robust standard errors in parentheses ***p < 0.01, **p < 0.05, *p < 0.1. The T-value is enclosed in parentheses.

To further explore how HEC policies impact specific dimensions of the IRC system, regressions are conducted on various dimensions of resilience, including economic, production, ecological, infrastructure, and social resilience, with results shown in columns (3) to (7) of Table 4. The findings reveal that HEC policies significantly enhance economic, ecological, and infrastructure resilience at the 1% significance level. However, the effects on production resilience and social resilience are not statistically significant, suggesting that while HEC policies boost economic, ecological, and infrastructure resilience, they do not significantly influence production or social organizational resilience.

Additionally, Fiscal Decentralization (FD) does not significantly affect IRC, though it positively impacts Urban Openness (UO), Government Macroeconomic Control capacity (GMC), and Financial Development level (FDL). Overall, the DID coefficients remain significant after controlling for city and year variables, with both adjusted and unadjusted coefficients indicating a strong model fit.

### 4.3 Mechanism analysis

Theoretical analyses have demonstrated that HEC policies are effective in enhancing environmental governance and promoting regional economic development (32, 68). To explore how HEC policies impact IRC, this study replaces traditional development variables with mechanism variables and tests them using a moderated effects model.

This study uses the Advancement of Industrial Structure (AIS) index, calculated for 180 cities in the Yangtze and Yellow River basins using data from the China Industrial Enterprises Database. Green technological innovation is also key to enhancing IRC. This is measured by the number of granted green invention patents, which better reflect actual R&D efforts and innovation value than patent applications (45, 89). The results, presented in Table 5, reveal significant relationships between green innovation, industrial structure, and the effects of HEC policies on IRC.

**Table 5:** Moderating effect test results.

Variable	(1)	(2)
	IRC	IRC
did	0.00287^**^	0.00263^**^
	[0.001]	[0.001]
AIS	0.00531^***^	
	[0.002]	
did_AIS	0.00052^*^	
	[0.000]	
GIL		−0.00128^*^
		[0.001]
did_GIL		0.00076^*^
		[0.000]
UO	−0.06816^**^	−0.05798^*^
	[0.033]	[0.034]
GMC	−0.01840^**^	−0.01931^**^
	[0.008]	[0.008]
FD	−0.00206	0.00055
	[0.004]	[0.004]
FDL	0.00447^**^	0.00647^***^
	[0.002]	[0.002]
Contr	0.07520^***^	0.08216^***^
	[0.004]	[0.005]
r2_within	0.01902	0.01423
*N*	2880.00000	2880.00000

Robust standard errors in parentheses *** *p* < 0.01, ** *p* < 0.05, * *p* < 0.1. Standard errors are in parentheses.

Table 5 (1) explores the moderating role of advanced industrial structures. Incorporating an interaction term between advanced industrial structure and HEC policy into the baseline regression model reveals that industrial structure significantly enhances IRC at the 5% significance level. This empirical evidence confirms Hypothesis 2, indicating that cities with more advanced industrial structures amplify the positive effects of HEC policies on IRC.

To further examine the moderating effect of green innovation, an interaction term between green innovation level and HEC policy is introduced into the benchmark regression model. The results, shown in column (2) of Table 5, indicate a positive relationship between this interaction term and IRC, also significant at the 5% level. This finding supports Hypothesis 3, demonstrating that higher levels of green innovation strengthen the positive impact of HEC policies on IRC, facilitating sustainable urban development.

### 4.4 Parallel trend test and analysis of the dynamic effects

This study employs a multi-period DID model, which assumes that both the experimental and control groups follow a similar trend of change before the policy implementation, satisfying the parallel trend assumption.

Therefore, this paper adopts the event study method, takes the city that implements the HEC policy for the first time as the experimental group, selects the previous year as the base period, and establishes the model shown in Equation 18 to estimate the dynamic effect of the HEC policy on the urban IRC index:


(18) $$\begin{array}{c} \mathrm{IR}C_{\mathrm{it}}=\beta_{0}+\beta_{k}\sum_{k=-4,k\neq -1}^{3}D_{i,t+k}+\text{γControl}\_\mathrm{Va}r_{\mathrm{it}}\\ +\text{CiFE}+\text{YearFE}+\varepsilon_{\mathrm{it}} \end{array}$$

Among them, $D_{i,t+k}$ represents the dummy variable for the relative time of each city implementing the HEC policy, while the other variables are defined as in the benchmark regression model. This paper excludes event occurrence points where k = −1 and uses the year before the implementation of the HEC policy as the base period year. The focus is on the core parameter $\beta_{k}$, which measures the impact of the k-th year of the HEC policy on urban IRC index.

**Figure 3:**
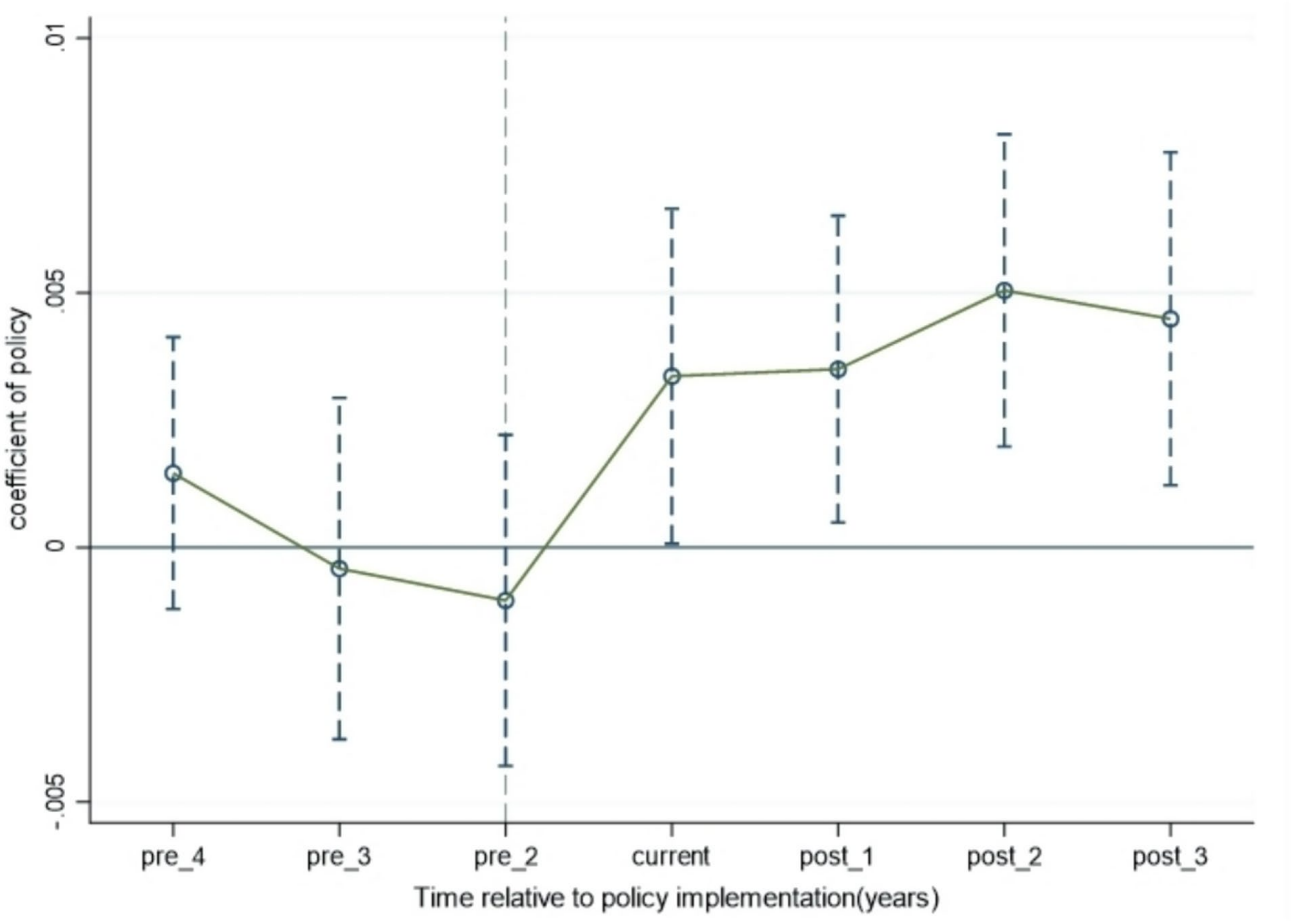
Parallel trend test results.

Figure 3 presents point estimates and 95% confidence intervals within a 7-year window (kϵ[—4, 3], k = —1). From Figure 3, it can be seen that before the implementation of the HEC policy, there was no significant difference in urban IRC index between the experimental group and the control group. After the policy was implemented, its positive effect on urban IRC index gradually became evident. However, it should be noted that the effect of horizontal ecological compensation policy on urban IRC index decreased in the third year after the implementation of the policy, indicating that the effect of horizontal ecological compensation policy on urban IRC index needs to be improved and long-term guarantee of the policy needs to be strengthened.

### 4.5 Robustness test

To comprehensively verify the robustness of the core findings, four distinct robustness tests are conducted in this section, each targeting different potential sources of bias or endogeneity.

#### 4.5.1 Placebo test to validate causal inference

Although this paper controls for various city characteristic variables in the quasi-natural experiment, some unobserved factors may still influence the evaluation of HEC policies. Due to the staggered implementation timelines of pilot cities in the multi-period DID analysis, a placebo test is necessary. This test involves randomly generating pseudo-treatment and pseudo-policy shock dummy variables by selecting random policy start times for each city in the sample.

**Figure 4:**
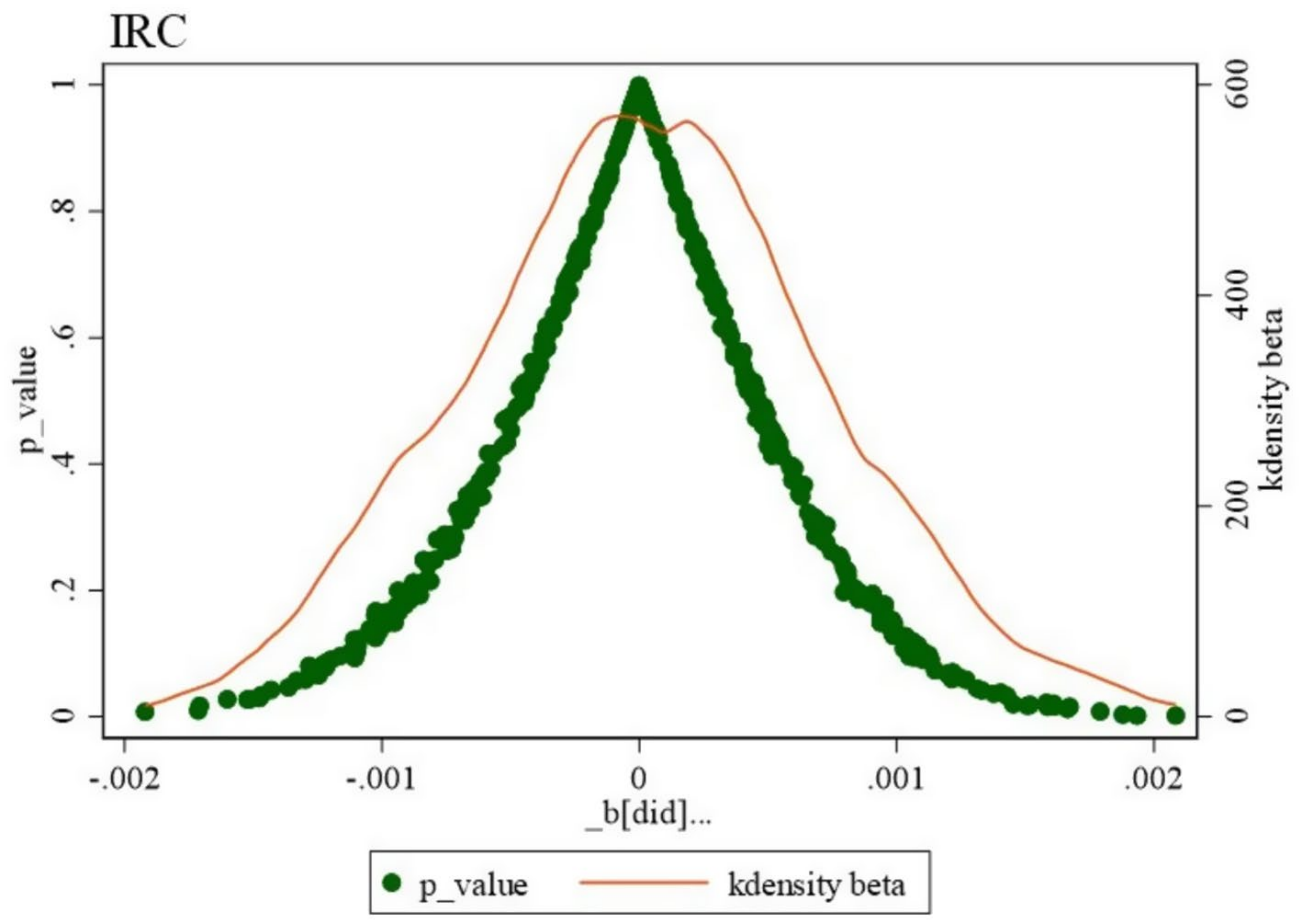
Placebo test.

To conduct the placebo test, this study randomly sampled 180 cities and time points without repeating the experimental cities or policy timelines. This process was repeated 500 times, generating 500 sets of random dummy variables $\mathrm{HEC}\_\text{Polic}y^{\text{random}}$, and the corresponding kernel density plots and *p*-value distributions are presented in Figure 4. The baseline regression coefficient in this study is 0.003, which is situated in the lower tail of the placebo test parameter distribution curve, with a *p*-value below 0.1, indicating a significant difference from the placebo results. In contrast, the *p*-values for most estimated coefficients are above 0.1, and the average regression coefficient from the random samples is close to zero. These findings confirm that the baseline regression of this study passes the placebo test, demonstrating the robustness of the evaluation results.

#### 4.5.2 Robustness testing via control variable adjustment

The data used in the regression analysis may contain inaccuracies due to measurement, input, or calculation errors, which could affect the robustness of the results. To address this, the model was re-estimated by reducing the control variables by 1 and 5%, respectively, with the regression outcomes presented in columns (3) and (4) of Table 6. The DID regression coefficients were 0.035 and 0.0030, both significant at the 1% level, indicating that the baseline regression results remain strong and reliable.

**Table 6:** Robustness test result.

Variable	PSM-DID		Reduced control variables		Delete municipalities
	Cross-section PSM	Yearly PSM	(1%)	(5%)	
	(1)	(2)	(3)	(4)	
did	0.0033**	0.0035**	0.0035***	0.0030***	0.0037***
	(2.3592)	(2.4057)	(3.3224)	(2.8672)	(3.4526)
X1	−0.1002*	−0.1185*	−0.0700**	−0.0954**	−0.0691**
	(−1.7815)	(−1.6820)	(−2.0433)	(−2.4683)	(−2.0771)
X2	−0.0157	−0.0125	−0.0166	−0.0298**	−0.0199***
	(−1.1124)	(−0.7548)	(−1.5895)	(−2.2446)	(−2.6225)
X3	−0.0026	−0.0046	−0.0008	−0.0030	−0.0016
	(−0.5037)	(−0.8300)	(−0.1929)	(−0.6491)	(−0.3699)
X4	0.0051	0.0053	0.0044**	0.0033	0.0066***
	(1.6407)	(1.6106)	(1.9895)	(1.4692)	(3.2292)
Year fix	YES	YES	YES	YES	YES
City fix	YES	YES	YES	YES	YES
Adj. *R*^2^	0.7631	0.7681	0.7896	0.7847	0.7867

Robust standard errors in parentheses *** *p* < 0.01, ** *p* < 0.05, * *p* < 0.1. The T-value is in parentheses.

#### 4.5.3 Exclusion of municipalities for robustness test

Given that municipalities are provincial-level administrative units with distinct administrative levels, policy resources, and urban scales compared to other cities, their inclusion could bias the assessment of IRC. To mitigate this, municipalities directly under the central government were excluded from the sample, and the results of this adjusted regression are shown in column (5) of Table 6. The DID coefficient of 0.0037, significant at the 1% level, further supports the robustness of the initial regression conclusions.

#### 4.5.4 Addressing sample selection bias using multi-period PSM-DID

While the DID approach isolates the average treatment effect of the pilot policy, the selection bias may still exist because the pilot HEC policy does not qualify as a strict natural experiment. To further test robustness, a multi-period PSM-DID model was employed. Given that PSM is suitable for cross-sectional data and DID for panel data, two approaches were adopted: 1. constructing a cross-sectional PSM by treating panel data as cross-sectional for matching, and 2. matching data period-by-period, as suggested by Böckerman and Ilmakunnas (92). Accordingly, this study sequentially applied the panel data transformation and the period-by-period matching methods for propensity score matching.

Columns (1) and (2) of Table 3 display the PSM-DID regression results across multiple time points using both methods. The results indicate that the DID coefficient remains significantly positive and closely aligns with the baseline findings, demonstrating that the impact of HEC policies on enhancing IRC is robust.

Additionally, subgroup heterogeneity analyses based on regional and developmental characteristics are presented in Section 4.6.3, which further reinforce the robustness and contextual relevance of the conclusions.

### 4.6 Heterogeneity analysis

Given China’s diverse regional characteristics, the effects of HEC policies may differ across cities with varying ecological vulnerability and development levels. This subsection conducts a heterogeneity analysis across three dimensions: urban hierarchy, geographical location, and administrative designation. The results provide deeper insights into the spatially differentiated impacts of HEC policies within the Yangtze and Yellow River basins.

#### 4.6.1 Urban hierarchy heterogeneity

The process of marketization varies significantly across cities, and this, combined with different policy-driven subsidies and city-specific characteristics, can influence the development of sustainable urban resilience. Following Wang (53), the sample cities are classified into two categories: central cities, including provincial capitals, sub-provincial cities, and the four municipalities, and peripheral cities, which encompass all other cities.

Table 7 presents the estimation results of how different city types affect IRC. The results reveal that HEC policies positively affect peripheral cities at the 1% level. However, their impact on central cities is not significant. This suggests that HEC policies help reduce regional disparities by supporting peripheral cities to catch up with more developed central cities. In contrast, they have minimal effects on central cities with higher development levels.

**Table 7:** Heterogeneous result of urban hierarchy.

Variable	(1)	(2)
	Central city	Non-central city
did	0.002	0.003***
	(0.40)	(3.24)
X1	−0.014	−0.051
	(−0.13)	(−1.41)
X2	−0.161**	−0.007
	(−2.15)	(−0.62)
X3	−0.011	0.002
	(−0.58)	(0.55)
X4	0.012*	0.004*
	(1.82)	(1.68)
*N*	336	2,544
*R* ^2^	0.733	0.807
Adj. *R*^2^	0.70	0.79

Robust standard errors in parentheses *** *p* < 0.01, ** *p* < 0.05, * *p* < 0.1. The T-value is in parentheses.

#### 4.6.2 Geographical location heterogeneity

To examine whether geographical differences impact the effectiveness of HEC policies on IRC, this study first divides 180 cities along the Yangtze River into upstream, midstream, and downstream clusters. This approach helps assess whether the initial implementation of HEC policies yields different outcomes across these catchment areas. As shown in Table 8, columns (1) to (3), the HEC policy significantly improves IRC in the upstream and midstream urban areas but shows no significant effect in the downstream regions. This disparity may be attributed to the lower economic development levels in the upstream and midstream regions, making them more responsive to policy-induced changes. In contrast, the downstream regions, characterized by a more advanced digital economy and higher development quality, exhibit less noticeable improvements under the same policy conditions.

**Table 8:** Heterogeneous result of location.

Variable	(1)	(2)	(3)	(4)	(5)	(6)
	Upper reaches	Middle reaches	Lower reaches	Eastern region	Middle region	Western region
did	0.004**	0.004**	−0.000	0.002	0.003**	0.004***
	(2.09)	(2.33)	(−0.24)	(0.61)	(2.15)	(2.68)
X1	0.007	−0.059	−0.141***	0.080	−0.083*	−0.030
	(0.07)	(−0.94)	(−2.77)	(0.93)	(−1.69)	(−0.38)
X2	0.011	−0.038	−0.041*	−0.034	−0.018	0.007
	(0.77)	(−1.48)	(−1.82)	(−1.23)	(−0.72)	(0.51)
X3	−0.002	−0.001	0.005	0.052***	−0.005	−0.001
	(−0.21)	(−0.17)	(0.64)	(3.85)	(−0.97)	(−0.10)
X4	−0.001	0.004	0.008**	0.004	−0.000	0.002
	(−0.18)	(0.89)	(2.04)	(0.68)	(−0.06)	(0.67)
Year fix	Yes	Yes	Yes	Yes	Yes	Yes
City fix	Yes	Yes	Yes	Yes	Yes	Yes
*N*	768	960	1,152	640	1,232	1,008
*R* ^2^	0.812	0.830	0.783	0.756	0.821	0.820
Adj. *R*^2^	0.79	0.81	0.76	0.73	0.81	0.80

Robust standard errors in parentheses *** *p* < 0.01, ** *p* < 0.05, * *p* < 0.1. The T-value is in parentheses.

#### 4.6.3 Administrative location heterogeneity

Furthermore, the study explores the regional impact of HEC policies by categorizing the 180 cities into eastern, central, and western regions based on their administrative locations. As shown in Table 8, columns (4) to (6), the results indicate that HEC policies significantly enhance IRC in the central and western regions, while the impact on the eastern region remains insignificant. Similar to the watershed findings, this regional difference can be explained by the eastern region’s resource abundance and economic efficiency, which make further significant improvements less attainable. Conversely, the central and western regions, being relatively less developed, experience more pronounced IRC advancements under the influence of HEC policies.

## 5 Results and discussion

This study employs the IRC index to evaluate the level of inclusive urban resilience in the Yangtze and Yellow River Basins from 2007 to 2022. The results reveal that the average IRC value for these regions is 0.081, indicating a generally low level of inclusive resilience with significant regional disparities, with downstream areas exhibiting significantly higher IRC values. The effects of HEC policies on local IRC levels were further assessed. Beyond assessing the marginal effects of HEC policies, the moderating roles of industrial structures and green innovation were also analyzed. The robustness of the results was validated using placebo tests, PSMDID, reduced-tail treatment, and exclusion of certain municipalities. Heterogeneity analysis further supported the robustness of these results. However, when the analysis was extended to downstream, eastern, and central cities, no significant empirical association between HEC policies and IRC was identified.

These results offer significant policy implications. First, the results suggest that ecological compensation mechanisms can contribute not only to environmental protection but also to inclusive and resilient urban growth, aligning with the objectives of Sustainable Development Goal 11. Notably, HEC policies positively influence IRC in upstream, central and western areas, and non-central cities, highlighting their potential to address spatial inequality in urban development. Second, HEC initiatives support low-carbon urban transformation by promoting cleaner industrial structures and regional cooperation. These efforts facilitate a transition toward sustainable urban economies and enhance resilience to climate-related risks. However, the observed regional differences underscore the need for context-sensitive policy design.

Despite these benefits, HEC policies also exhibit inherent limitations. Fiscal burdens may disproportionately affect underdeveloped areas, particularly when compensation standards are misaligned with local opportunity costs. Overreliance on central government transfers may also weaken the intrinsic motivation of recipient regions to pursue environmental reforms. Additionally, policy enforcement effectiveness remains uneven, depending heavily on local governance capacity and alignment with the performance evaluation system for officials.

To enhance the impact and equity of HEC policies, future reforms could consider optimizing the design of compensation mechanisms, ensuring alignment with local economic conditions and ecological values. Interregional coordination platforms can be established to better manage watershed-level governance, particularly in addressing transboundary water conflicts. Stronger integration with national ecological strategies—such as the “dual carbon” goals, ecological civilization reform, and the Yangtze River Protection Law—would increase policy coherence. Furthermore, differentiated policy guidance should be developed based on regional climate risk profiles and stages of economic development, while financial support for green innovation should be expanded to empower local governments in achieving both environmental and resilience targets.

Several limitations of this study warrant further discussion. Although the analysis focuses on the HEC policies due to their relevance to watershed governance and urban resilience (33, 43), other environmental factors such as air pollution remain underexplored. Recent research highlights the crucial role of air pollution regulation in China’s ecological governance (11, 90) which should be incorporated into future research frameworks. Moreover, challenges specific to river basin governance, such as interprovincial water disputes and hydrological variability under climate change, were not fully incorporated in this study. These are especially relevant in China’s major river systems and should be prioritized in future work.

Further research is should also aim to establish a more comprehensive and standardized framework for measuring inclusive urban resilience. Currently, there is no unified national standard, and indicators such as government capacity, public risk response, firm-level emissions, and overall IRC levels are often applied without clear weighting guidance, especially in county-level cities. Future research could address these gaps through field surveys and qualitative assessments.

While additional inquiry remains necessary, it is hoped that this study provides a foundational contribution to the growing literature on ecological compensation policies and their role in fostering resilient and inclusive urban development in China.

## Data Availability

The datasets presented in this study can be found in online repositories. The names of the repository/repositories and accession number(s) can be found in the article/Supplementary material.

## References

[1] Leal W Mbah MF Dinis MAP Trevisan LV de Lange D Mishra A. The role of artificial intelligence in the implementation of the un sustainable development goal 11: fostering sustainable cities and communities. Cities. (2024) 150:105021. doi: 10.1016/j.cities.2024.105021, PMID: 40625364

[2] Albert C Rufat S Kuhlicke C. Five principles for making cities climate-resilient. Nature. (2021) 596:486–6. doi: 10.1038/d41586-021-02309-9, PMID: 34429541

[3] Finn D Chandrasekhar D Xiao Y. A region recovers: planning for resilience after superstorm Sandy. J Plan Educ Res. (2023) 43:136–49. doi: 10.1177/0739456x19864145

[4] Wong CY Wang IK. Resistant, path creation, or resilient? An empirical study of 87 innovative cities worldwide. Seoul J Econ. (2023) 36:165–91. doi: 10.22904/sje.2023.36.2.001

[5] Zhang R Yan W Shaw R. Land use planning and conservation policy in the Tokyo metropolitan area In: Yan W Galloway W Shaw R, editors. Resilient and adaptive Tokyo: Towards sustainable urbanization in perspective of food-energy-water Nexus. Springer Nature Singapore: Singapore (2024)

[6] Wang WD Zhang L Peng T. Evaluation of a safe resilient City: a comparison of Hangzhou and Shaoxing. China Sustain Cities Soc. (2023) 98:104798. doi: 10.1016/j.scs.2023.104798, PMID: 40625364

[7] Chen Y Dou S Xu D. The effectiveness of eco-compensation in environmental protection -a hybrid of the government and market. J Environ Manag. (2021) 280:111840. doi: 10.1016/j.jenvman.2020.111840, PMID: 33360550

[8] Li X Li PJ Wang D Wang YQ. Assessment of temporal and spatial variations in water quality using multivariate statistical methods: a case study of the Xin'anjiang river, China. Front Environ Sci Eng. (2014) 8:895–904. doi: 10.1007/s11783-014-0736-z

[9] Ren YS Lu L Zhang HM Chen HF Zhu DC. Residents' willingness to pay for ecosystem services and its influencing factors: a study of the xin'an river basin. J Clean Prod. (2020) 268:122301. doi: 10.1016/j.jclepro.2020.122301

[10] Yu HJ Xie W Yang L Du AS Almeida C Wang YT. From payments for ecosystem services to eco-compensation: conceptual change or paradigm shift? Sci Total Environ. (2020) 700:134627. doi: 10.1016/j.scitotenv.2019.134627, PMID: 31693962

[11] Jiang SR Tan X Hu PQ Wang Y Shi L Ma Z. Air pollution and economic growth under local government competition: evidence from China, 2007-2016. J Clean Prod. (2022) 334:130231. doi: 10.1016/j.jclepro.2021.130231

[12] Lin S Zhang S Yang Q Cai Y Li X Ren Z. Rapid urbanization and global warming significantly impact tidal dynamics in the Pearl River estuary, China. Watershed Ecol Environment. (2023) 5:100–7. doi: 10.1016/j.wsee.2023.03.001, PMID: 40625364

[13] Pagiola S Arcenas A Platais G. Can payments for environmental services help reduce poverty? An exploration of the issues and the evidence to date from Latin America. World Dev. (2005) 33:237–53. doi: 10.1016/j.worlddev.2004.07.011

[14] Tu Y Chen B Yu L Song Y Wu S Li M. Raveling the nexus between urban expansion and cropland loss in China. Landsc Ecol. (2023) 38:1869–84. doi: 10.1007/s10980-023-01653-7

[15] Zhang HZ He LY Zhang ZX. Can transverse eco-compensation mechanism correct resource misallocation in watershed environmental governance? A cost-benefit analysis of the pilot project of xin'an river in China. Environ Res Econ. (2023) 84:947–73. doi: 10.1007/s10640-022-00743-5

[16] Zhu Y Xia Y. Industrial agglomeration and environmental pollution: evidence from China under new urbanization. Energy Environ. (2018) 30:1010–26. doi: 10.1177/0958305X18802784, PMID: 40620826

[17] Hardoy J Gencer E Winograd M. Participatory planning for climate resilient and inclusive urban development in Dosquebradas, Santa Ana and Santa tome. Environ Urban. (2019) 31:33–52. doi: 10.1177/0956247819825539

[18] Nederhand J Avelino F Awad I De Jong P Duijn M Edelenbos J. Reclaiming the city from an urban vitalism perspective: critically reflecting smart, inclusive, resilient and sustainable just city labels. Cities. (2023) 137:104257. doi: 10.1016/j.cities.2023.104257

[19] DuPuis EM Greenberg M. The right to the resilient city: progressive politics and the green growth machine in new York City. J Environ Stud Sci. (2019) 9:352–63. doi: 10.1007/s13412-019-0538-5

[20] Ferreira HS Robinson N Serraglio DA. Climate migration and resilient cities: a new urban agenda for sustainable development. Revista De Direito Da Cidade-City Law. (2019) 11:304–46. doi: 10.12957/rdc.2019.38103

[21] Zoomers A van Noorloos F Otsuki K Steel G van Westen G. The rush for land in an urbanizing world: from land grabbing toward developing safe, resilient, and sustainable cities and landscapes. World Dev. (2017) 92:242–52. doi: 10.1016/j.worlddev.2016.11.016

[22] Diaz-Sarachaga JM Jato-Espino D. Development and application of a new resilient, sustainable, safe and inclusive community rating system (Ressicom). J Clean Prod. (2019) 207:971–9. doi: 10.1016/j.jclepro.2018.10.061

[23] Wahba SN. Can cities bounce back better from Covid-19? Reflections from emerging post-pandemic recovery plans and trade-offs. Environ Urban. (2022) 34:481–96. doi: 10.1177/09562478221102867

[24] Cheek W Chmutina K. Measuring resilience in the Assumed City. Int J Disaster Risk Sci. (2022) 13:317–29. doi: 10.1007/s13753-022-00410-9

[25] Wojewnik-Filipkowska A Gierusz-Matkowska A Krauze-Maslankowskac P. Fundamental power of the city - a proposition of a new paradigm and index for city development. Cities. (2024) 144:104630. doi: 10.1016/j.cities.2023.104630

[26] Giles-Corti B Foster S Lynch B Lowe M. What are the lessons from Covid-19 for creating healthy, sustainable, resilient future cities? NPJ Urban Sustain. (2023) 3:29. doi: 10.1038/s42949-023-00107-y, PMID: 37305613 PMC10236403

[27] Pauleit S Ambrose-Oji B Andersson E Anton B Buijs A Haase D. Advancing urban green infrastructure in Europe: outcomes and reflections from the green surge project. Urban For Urban Green. (2019) 40:4–16. doi: 10.1016/j.ufug.2018.10.006

[28] Peters V Wang JT Sanders M. Resilience to extreme weather events and local financial structure of prefecture-level cities in China [J]. Clim Chang. (2023) 176:125. doi: 10.1007/s10584-023-03599-w

[29] Schintler LA McNeely CL. Artificial intelligence, institutions, and resilience: prospects and provocations for cities. J Urban Manage. (2022) 11:256–68. doi: 10.1016/j.jum.2022.05.004

[30] Wang D Chen SW. The effect of pilot climate-resilient city policies on urban climate resilience: evidence from quasi-natural experiments. Cities. (2024) 153:105316. doi: 10.1016/j.cities.2024.105316

[31] Zhou Q Qiao YR Zhang H Zhou S. How does college scale affect urban resilience? Spatiotemporal evidence from China. Sustain Cities Soc. (2022) 85:104084. doi: 10.1016/j.scs.2022.104084

[32] Jin ST Jiang AL Bao BF. Can China's transfer payment in key ecological function areas reduce the carbon intensity?-quasi-natural experimental evidence from Jiangxi, China. Ecol Indic. (2023) 154:110537. doi: 10.1016/j.ecolind.2023.110537, PMID: 40625364

[33] Li N Cheng CG Mou HS Deng MJ Tang DS Yang DY. Application of eco-compensation to control transboundary water pollution in water diversion projects: the case of the Heihe River transfer project in China. Ecol Indic. (2024) 158:111326. doi: 10.1016/j.ecolind.2023.111326, PMID: 40625364

[34] Wan L Zheng QQ Wu J Wei ZY Wang SY. How does the ecological compensation mechanism adjust the industrial structure? Evidence from China. J Environ Manag. (2022) 301:113839. doi: 10.1016/j.jenvman.2021.113839, PMID: 34592663

[35] Hao Y Xu L Guo Y Wu H. The inducing factors of environmental emergencies: do environmental decentralization and regional corruption matter? J Environ Manag. (2021) 302:114098. doi: 10.1016/j.jenvman.2021.114098, PMID: 34794054

[36] Li HL Wen ZM Wan YM Hu JX. How does the horizontal watershed ecological compensation mechanism effect regional economy?- a county level empirical study on xin'an ' an river basin, China. Ecol Indic. (2024) 166:112506. doi: 10.1016/j.ecolind.2024.112506, PMID: 40625364

[37] Cao RF Zhang AL Wen LJ. Trans-regional compensation mechanism under imbalanced land development: from the local government economic welfare perspective. Habitat Int. (2018) 77:56–63. doi: 10.1016/j.habitatint.2018.04.001

[38] Li J Ye ZW Zhuang J Okada N Huang L Han GY. Changes of public risk perception in China: 2008-2018. Sci Total Environ. (2021) 799:149453. doi: 10.1016/j.scitotenv.2021.149453, PMID: 34388887

[39] Mian Y Zeyu X Chusheng Y. Beyond the environmental Kuznets curve: an empirical study taking China's poverty alleviation campaign as a quasi-experiment. Soc Sci China. (2023) 44:98–128. doi: 10.1080/02529203.2023.2192089

[40] Qin B Yu Y Ge L Yang L Guo Y. Does eco-compensation alleviate rural poverty? New Evidence from National key Ecological Function Areas in China. Int J Environ Res Public Health. (2022) 19:10899. doi: 10.3390/ijerph191710899 36078613 10.3390/ijerph191710899 PMC9518322

[41] Gao JX Duan CZ Song JB Ma XR Wang YP. Two-stage and three-party transboundary watershed management based on valuation adjustment mechanism (Vam) agreement. Water Resour Manag. (2023) 37:3343–75. doi: 10.1007/s11269-023-03505-0

[42] Liu YW Yuan L. Study on the influencing factors and profitability of horizontal ecological compensation mechanism in Yellow River Basin of China. Environ Sci Pollut Res. (2023) 30:87353–67. doi: 10.1007/s11356-023-28243-z, PMID: 37422555

[43] Yang Y Zhang YY Yang H Yang FY. Horizontal ecological compensation as a tool for sustainable development of urban agglomerations: exploration of the realization mechanism of Guanzhong plain urban agglomeration in China. Environ Sci Pol. (2022) 137:301–13. doi: 10.1016/j.envsci.2022.09.004

[44] Quan TS Zhang H Li J Lu BQ. Horizontal ecological compensation mechanism and green low-carbon development in river basins: evidence from xin'an river basin. Environ Sci Pollut Res. (2023) 30:88463–80. doi: 10.1007/s11356-023-28679-3, PMID: 37434059

[45] Yang QY. Do vertical ecological compensation policies promote green economic development: a case study of the transfer payments policy for China's National key Ecological Function Zones. Econ Syst. (2023) 47:101125. doi: 10.1016/j.ecosys.2023.101125

[46] Gong J Du HY Wang Z. Analysis of the influences of ecological compensation projects on transfer employment of rural labor from the perspective of capability. Land. (2022) 11:1464. doi: 10.3390/land11091464

[47] Ju F Zhou JJ Jiang K. Evolution of stakeholders' behavioral strategies in the ecological compensation mechanism for poverty alleviation. Res Conservation Recycling. (2022) 176:105915. doi: 10.1016/j.resconrec.2021.105915

[48] Selva GV Pauli N Kim MK Clifton J. Can environmental compensation contribute to socially equitable conservation? The case of an ecological fiscal transfer in the Brazilian Atlantic forest. Local Environ. (2019) 24:931–48. doi: 10.1080/13549839.2019.1663800

[49] Wu L Jin LS. How eco-compensation contribute to poverty reduction: a perspective from different income group of rural households in Guizhou. China J Cleaner Produc. (2020) 275:122962. doi: 10.1016/j.jclepro.2020.122962, PMID: 40625364

[50] Barnthouse LW Stahl RG Jr. Quantifying natural resource injuries and ecological service reductions: challenges and opportunities. Environ Manag. (2002) 30:1–12. doi: 10.1007/s00267-001-2447-z, PMID: 12053235

[51] Gao Y Yu L. Understanding the impacts of ecological compensation policies on energy poverty: insights from forest communities in Zhejiang. China Land Use Policy. (2024) 142:107144. doi: 10.1016/j.landusepol.2024.107144, PMID: 40625364

[52] Lei M Yuan XY Yao XY. Synthesize dual goals: a study on China's ecological poverty alleviation system. J Integr Agric. (2021) 20:1042–59. doi: 10.1016/s2095-3119(21)63635-3

[53] Wang HS Yang GQ Ouyang X Tand ZY Long XF Yue ZY. Horizontal ecological compensation mechanism and technological progress: theory and empirical study of xin'an river ecological compensation gambling agreement. J Environ Plan Manag. (2023) 66:501–23. doi: 10.1080/09640568.2021.1990030

[54] Ding JP Chen LX Deng MH Chen JF. A differential game for basin ecological compensation mechanism based on cross-regional government-enterprise cooperation. J Clean Prod. (2022) 362:132335. doi: 10.1016/j.jclepro.2022.132335

[55] Wu M Cao X. Greening the career incentive structure for local officials in China: does less pollution increase the chances of promotion for Chinese local leaders? J Environ Econ Manag. (2021) 107:102440. doi: 10.1016/j.jeem.2021.102440, PMID: 40625364

[56] Liu W Tong J Yue XH. How does environmental regulation affect industrial transformation? A study based on the methodology of policy simulation. Math Probl Eng. (2016) 2016:1–10. doi: 10.1155/2016/2405624, PMID: 40435296

[57] Cansino JM Carril-Cacia F Molina-Parrado JC Roman-Collado R. Do environmental regulations matter on Spanish foreign investment? A multisectorial approach. Environ Sci Pollut Res. (2021) 28:57781–97. doi: 10.1007/s11356-021-14635-6, PMID: 34100206

[58] Cole MA. Trade, the pollution haven hypothesis and the environmental kuznets curve: examining the linkages. Ecol Econ. (2004) 48:71–81. doi: 10.1016/j.ecolecon.2003.09.007

[59] Demirel P Iatridis K Kesidou E. The impact of regulatory complexity upon self-regulation: evidence from the adoption and certification of environmental management systems. J Environ Manag. (2018) 207:80–91. doi: 10.1016/j.jenvman.2017.11.019, PMID: 29154011

[60] El-Zayat H Ibraheem G Kandil S. The response of industry to environmental regulations in Alexandria. Egypt J Environ Manage. (2006) 79:207–14. doi: 10.1016/j.jenvman.2005.07.002, PMID: 16202506

[61] Lian TH Ma TY Cao J Wu Y. The effects of environmental regulation on the industrial location of China's manufacturing. Nat Hazards. (2016) 80:1381–403. doi: 10.1007/s11069-015-2008-z

[62] Liu LJ Jiang JY Bian JC Liu YZ Lin GH Yin YK. Are environmental regulations holding back industrial growth? Evidence from China. J Clean Prod. (2021) 306:127007. doi: 10.1016/j.jclepro.2021.127007

[63] Zhang HM Zhu ZS Fan YJ. The impact of environmental regulation on the coordinated development of environment and economy in China. Nat Hazards. (2018) 91:473–89. doi: 10.1007/s11069-017-3137-3

[64] Dai LX Li ST. The impact of environmental regulatory instruments on firm investment efficiency: evidence from Chinese listed heavy polluters. Pol J Environ Stud. (2023) 32:4541–54. doi: 10.15244/pjoes/168903

[65] Zhang HY Chen HH Lao KS Ren ZY. The impacts of resource endowment, and environmental regulations on sustainability-empirical evidence based on data from renewable energy enterprises. Energies. (2022) 15:4678. doi: 10.3390/en15134678

[66] Wang LH Wang Z Ma YT. Heterogeneous environmental regulation and industrial structure upgrading: evidence from China. Environ Sci Pollut Res. (2022) 29:13369–85. doi: 10.1007/s11356-021-16591-7, PMID: 34591249

[67] Ge L Zhao HX Yang JY Yu JY He TY. Green finance, technological progress, and ecological performance-evidence from 30 provinces in China. Environ Sci Pollut Res. (2022) 29:66295–314. doi: 10.1007/s11356-022-20501-w, PMID: 35501430

[68] Gao D Li G Li Y Gao KX. Does Fdi improve green total factor energy efficiency under heterogeneous environmental regulation? Evidence from China. Environ Sci Pollut Res. (2022) 29:25665–78. doi: 10.1007/s11356-021-17771-1, PMID: 34845639

[69] Xu XY Hou P Liu Y. The impact of heterogeneous environmental regulations on the technology innovation of urban green energy: a study based on the panel threshold model. Green Finance. (2022) 4:115–36. doi: 10.3934/gf.2022006

[70] Jiang YJ Wu QR Brenya R Wang K. Environmental decentralization, environmental regulation, and green technology innovation: evidence based on China. Environ Sci Pollut Res. (2023) 30:28305–20. doi: 10.1007/s11356-022-23935-4, PMID: 36399298

[71] Yang ZQ Liu PY Luo LF. How does environmental regulation affect corporate green innovation: a comparative study between voluntary and mandatory environmental regulations. J Comp Policy Anal. (2024) 26:130–58. doi: 10.1080/13876988.2024.2328602, PMID: 40475060

[72] Ju KY Zhou DJ Wang QW Zhou DQ Wei XZ. What comes after picking pollution intensive low-hanging fruits? Transfer direction of environmental regulation in China. J Clean Prod. (2020) 258:120405. doi: 10.1016/j.jclepro.2020.120405

[73] Li SQ Shen JQ Sun FH Jia YZ Han HK. Quantitative evaluation of ecological compensation policies for the watershed in China: based on the improved policy modeling consistency index. Environ Sci Pollut Res. (2022) 29:66659–74. doi: 10.1007/s11356-022-20503-8, PMID: 35508855

[74] Zhang WEI Pagiola S. Assessing the potential for synergies in the implementation of payments for environmental services programmes: an empirical analysis of Costa Rica. Environ Conserv. (2011) 38:406–16. doi: 10.1017/S0376892911000555

[75] Lee HC Chang CT. Comparative analysis of Mcdm methods for ranking renewable energy sources in Taiwan. Renew Sustain Energy Rev. (2018) 92:883–96. doi: 10.1016/j.rser.2018.05.007

[76] Li DF. Topsis-based nonlinear-programming methodology for multiattribute decision making with interval-valued intuitionistic fuzzy sets. IEEE Trans Fuzzy Syst. (2010) 18:299–311. doi: 10.1109/tfuzz.2010.2041009, PMID: 40567712

[77] Tang D Li J Zhao Z Boamah V Lansana DD. The influence of industrial structure transformation on urban resilience based on 110 prefecture-level cities in the Yangtze River. Sustain Cities Soc. (2023) 96:104621. doi: 10.1016/j.scs.2023.104621

[78] Parker G Bhandari R Jain S Kaur S Arslan B Sharma D. Resilient Cities Index 2023, Economist Impact. (2023), Available at: https://impact.economist.com/projects/resilient-cities/ (Accessed May 17, 2025).

[79] Ribeiro PJG Gonçalves LAPJ. Urban resilience: a conceptual framework. Sustain Cities Soc. (2019) 50:101625. doi: 10.1016/j.scs.2019.101625

[80] Xun XL Yuan YB. Research on the urban resilience evaluation with hybrid multiple attribute Topsis method: an example in China. Nat Hazards. (2020) 103:557–77. doi: 10.1007/s11069-020-04000-0, PMID: 32412523 PMC7220654

[81] Burton CG. A validation of metrics for community resilience to natural hazards and disasters using the recovery from hurricane katrina as a case study. Ann Assoc Am Geogr. (2015) 105:67–86. doi: 10.1080/00045608.2014.960039

[82] Sharifi A. Resilient urban forms: a macro-scale analysis. Cities. (2019) 85:1–14. doi: 10.1016/j.cities.2018.11.023

[83] Ouyang M Dueñas-Osorio L Min X. A three-stage resilience analysis framework for urban infrastructure systems. Struct Saf. (2012) 36-37:23–31. doi: 10.1016/j.strusafe.2011.12.004

[84] Zhang XW Song J Peng J Wu JS. Landslides-oriented urban disaster resilience assessment-a case study in Shenzhen. China Sci Total Environ. (2019) 661:95–106. doi: 10.1016/j.scitotenv.2018.12.074, PMID: 30665136

[85] Li T Singh RK Pandey R Liu H Cui L Xu Z. Enhancing sustainable livelihoods in the three Rivers headwater region: a geospatial and obstacles context. Ecol Indic. (2023) 156:111134. doi: 10.1016/j.ecolind.2023.111134

[86] Xiang X Zhou YX. How does human capital promote industrial structure upgrading: an empirical study based on Chinese provincial panel data. Transform Bus Econ. (2022) 21:104–19.

[87] Dong MF Wang G Han XF. Artificial intelligence, industrial structure optimization, and Co2 emissions. Environ Sci Pollut Res. (2023) 30:108757–73. doi: 10.1007/s11356-023-29859-x, PMID: 37752399

[88] Abedini A Aram F Khalili A Mirzaei E. Recognition and evaluating the indicators of urban resilient by using the network analysis process. Urban. Science. (2022) 6:31. doi: 10.3390/urbansci6020031, PMID: 40607831

[89] Liu YQ Zhu JL Li EY Meng ZY Song Y. Environmental regulation, green technological innovation, and eco-efficiency: the case of Yangtze river economic belt in China. Technol Forecast Soc Chang. (2020) 155:119993. doi: 10.1016/j.techfore.2020.119993

[90] Weng SM Tao WL Cui LB. Can ecological compensation reduce air pollution? New evidence from resource-based cities in China. Energy Sources. (2024) 19:2351803. doi: 10.1080/15567249.2024.2351803

[91] Li Z Liu L Ye Y. Can urban agglomeration policy enhance comprehensive urban resilience? Evidence from China[J]. Nat Hazards Rev. (2024) 25:04024007. doi: 10.1061/NHREFO.NHENG-2001

[92] Böckerman P Ilmakunnas P. Unemployment and self-assessed health: evidence from panel data. Health Econ. (2009) 18:161–79. doi: 10.1002/hec.1361, PMID: 18536002

[93] Wang KG Zheng HH Zhao XY Sang ZT Yan WZ Cai ZY. Landscape ecological risk assessment of the Hailar River basin based on ecosystem services in China. Ecol Indic. (2023) 147:109795. doi: 10.1016/j.ecolind.2022.109795

