# Supplementary material for: Horizontal ecological compensation and urban resilience: mechanisms of low-carbon transformation
Source: Front Public Health. 2025 Sep 12;13:1583074. doi: 10.3389/fpubh.2025.1583074 (PMC12464017; doi:10.3389/fpubh.2025.1583074)
Supplement: Supplementary file 1 [file Table_1.DOCX]

| **State** | **City name** |
| --- | --- |
| **Shanghai** | **Shanghai** |
| **Jiangsu** | Nanjing |
| **Jiangsu** | Wuxi |
| **Jiangsu** | Xuzhou |
| **Jiangsu** | Changzhou |
| **Jiangsu** | Suzhou |
| **Jiangsu** | Nantong |
| **Jiangsu** | Huaian |
| **Jiangsu** | Yancheng |
| **Jiangsu** | Yangzhou |
| **Jiangsu** | Taizhou |
| **Jiangsu** | Suqian |
| **Zhejiang** | Hangzhou |
| **Zhejiang** | Ningbo |
| **Zhejiang** | Wenzhou |
| **Zhejiang** | Jiaxing |
| **Zhejiang** | Huzhou |
| **Zhejiang** | Jinhua |
| **Zhejiang** | Quzhou |
| **Zhejiang** | Zhoushan |
| **Zhejiang** | Taizhou |
| **An hui** | Hefei |
| **An hui** | Wuhu |
| **An hui** | Huainan |
| **An hui** | Huaibei |
| **An hui** | Anqing |
| **An hui** | Huangshan |
| **An hui** | Chuzhou |
| **An hui** | Fuyang |
| **An hui** | Suzhou |
| **An hui** | Bozhou |
| **Jiangxi** | Nanchang |
| **Jiangxi** | Jindezhen |
| **Jiangxi** | Pingxiang |
| **Jiangxi** | Yingtan |
| **Jiangxi** | Ganzhou |
| **Jiangxi** | Yichun |
| **Hubei** | Wuhan |
| **Hubei** | Huangshi |
| **Hubei** | Shiyan |
| **Hubei** | Yichang |
| **Hubei** | Xiangyang |
| **Hubei** | Ezhou |
| **Hubei** | Jingmen |
| **Hubei** | Xiaogan |
| **Hubei** | Jingzhou |
| **Hubei** | Huanggang |
| **Hunan** | Changsha |
| **Hunan** | Zhuzhou |
| **Hunan** | Xiangtan |
| **Hunan** | Hengyang |
| **Hunan** | Shaoyang |
| **Hunan** | Yueyang |
| **Hunan** | Yiyang |
| **Hunan** | Binzhou |
| **Hunan** | Yongzhou |
| **Hunan** | Huaihua |
| **Hunan** | Loudi |
| **Chongqing** | **Chongqing** |
| **Sichuan** | Chengdu |
| **Sichuan** | Zigong |
| **Sichuan** | Panzhihua |
| **Sichuan** | Luzhou |
| **Sichuan** | Deyang |
| **Sichuan** | Mianyang |
| **Sichuan** | Guangyuan |
| **Sichuan** | Suining |
| **Sichuan** | Neijiang |
| **Sichuan** | Leshan |
| **Sichuan** | Nanchong |
| **Sichuan** | Meishan |
| **Sichuan** | Yibing |
| **Sichuan** | Guangan |
| **Sichuan** | Dazhou |
| **Sichuan** | Bazhong |
| **Sichuan** | Ziyang |
| **Guizhou** | Guiyang |
| **Guizhou** | Liupanshui |
| **Guizhou** | Zunyi |
| **Guizhou** | Anshun |
| **Yunnan** | Kunming |
| **Yunnan** | Qujing |
| **Yunnan** | Yuxi |
| **Yunnan** | Baoshan |
| **Yunnan** | Zhaotong |
| **Yunnan** | Lijiang |
| **Yunnan** | Linchang |
